# Unmet supportive care needs among head and neck cancer survivors beyond 5 years after diagnosis: a multinational cohort study

**DOI:** 10.1016/j.lanepe.2025.101495

**Published:** 2025-10-16

**Authors:** Femke Jansen, Simone EJ. Eerenstein, Katherine J. Taylor, Cecilie D. Amdal, Kristin Bjordal, Guro L. Astrup, Bente B. Herlofson, Fréderic Duprez, Ricardo R. Gama, Alexandre A. Jacinto, Eva Hammerlid, Melissa Scricciolo, Giuseppe Fanetti, Orlando Guntinas-Lichius, Johanna Inhestern, Tatiana Dragan, Alexander Fabian, Andreas Boehm, Ulrike Wöhner, Naomi Kiyota, Maximilian Krüger, Pierluigi Bonomo, Monica Pinto, Sandra Nuyts, Joaquim Castro Silva, Carmen Stromberger, Pol Specenier, Francesco Tramacere, Ayman Bushnak, Pietro Perotti, Michaela Plath, Alberto Paderno, Noa Stempler, Antonia Kanellopoulou, Susanne Singer, Irma M. Verdonck-de Leeuw

**Affiliations:** a Department of Otolaryngology-Head and Neck Surgery, Amsterdam UMC, Location Vrije Universiteit, Amsterdam, The Netherlands; b Cancer Center Amsterdam, Treatment and Quality of Life, Amsterdam, The Netherlands; c Institute of Medical Biostatistics, Epidemiology, and Informatics, University Medical Centre Mainz, Mainz, Germany; d Department of Oncology, Oslo University Hospital, Oslo, Norway; e Research Support Services, Oslo University Hospital, Oslo, Norway; f Faculty of Medicine, University of Oslo, Oslo, Norway; g Faculty of Dentistry, University of Oslo, Oslo, Norway; h Unit of Oral and Maxillofacial Surgery, Department of Otorhinolaryngology, Oslo University Hospital, Oslo, Norway; i Department of Radiotherapy-Oncology, Ghent University Hospital, Faculty of Medicine and Health Sciences—Human Structure and Repair, Ghent University, Ghent, Belgium; j Department of Head and Neck Surgery, Barretos Cancer Hospital, Barretos, Brazil; k Department of Radiation Oncology, Barretos Cancer Hospital, Barretos, Brazil; l Department of Otorhinolaryngology-Head and Neck Surgery, Institute of Clinical Sciences, Sahlgrenska Academy at University of Gothenburg, Sahlgrenska University Hospital, Gothenburg, Sweden; m Radiation Oncology Division, Clinical Oncology Department, Ospedale dell'Angelo, Venice, Italy; n Division of Radiation Oncology, Centro di Riferimento Oncologico di Aviano (CRO) IRCCS, Aviano, Italy; o Department of Otorhinolaryngology, Jena University Hospital, Jena, Germany; p Department of Otorhinolaryngology, Oberhavel Kliniken Hennigsdorf, Hennigsdorf, Germany; q Department of Radiation Oncology, Head and Neck Unit, Institut Jules Bordet, Université Libre de Bruxelles, Brussels, Belgium; r Department of Radiation Oncology, University Hospital Schleswig-Holstein, Kiel, Germany; s Department of Otorhinolaryngology, St. Georg Hospital, Leipzig, Germany; t Cancer Centre, Kobe University Hospital, Kobe, Japan; u Department of Oral and Maxillofacial Surgery—Plastic Surgery, University Medical Centre Mainz, Mainz, Germany; v Radiation Oncology, Azienda Ospedaliero-Universitaria Careggi, Florence, Italy; w Rehabilitation Medicine Unit, Istituto Nazionale Tumori—IRCCS—Fondazione G. Pascale, Naples, Italy; x Laboratory of Experimental Radiotherapy, Department of Oncology, KU Leuven, Leuven, Belgium; y Department of Radiation Oncology, Leuven Cancer Institute, University Hospitals Leuven, Leuven, Belgium; z Department of Otolaryngology, Head and Neck Surgery, Instituto Português de Oncologia Francisco Gentil Do Porto, Porto, Portugal; aa Department of Radiation Oncology, Charité-Universitätsmedizin Berlin, Corporate Member of Freie Universität Berlin, Humboldt-Universität zu Berlin, Berlin, Germany; ab Berlin Institute of Health, Berlin, Germany; ac Department of Oncology, Antwerp University Hospital, Edegem, Belgium; ad Department of Radiation Oncology, Azienda Sanitaria Locale, Brindisi, Italy; ae Department of Otorhinolaryngology, University Hospital Gießenund Marburg, Giessen, Germany; af Department of Otorhinolaryngology—Head and Neck Surgery, “S·Chiara” Hospital, Azienda Provinciale Per I Servizi Sanitari (APSS), Trento, Italy; ag Department of Otorhinolaryngology, Head and Neck Surgery, University Hospital Heidelberg, Heidelberg, Germany; ah Department of Otorhinolaryngology—Head and Neck Surgery, ASST Spedali Civili of Brescia, University of Brescia, Brescia, Italy; ai Oral Medicine Unit, Sheba Medical Center, Tel Hashomer, Israel; aj Orofacial Pain Clinic, School of Dentistry, National and Kapodistrian University of Athens, Athens, Greece; ak Division of Quality of Life in Oncology, Comprehensive Cancer Center Mecklenburg-Vorpommern, University Medical Center Rostock, Germany; al Department of Clinical, Neuro and Developmental Psychology, Vrije Universiteit Amsterdam, Amsterdam, The Netherlands

**Keywords:** Head and neck cancer, Supportive care, Europe, Multinational, Supportive care needs, Healthcare system

## Abstract

**Background:**

This study investigates unmet supportive care needs (SCNs) among head and neck cancer (HNC) survivors beyond 5 years after diagnosis and examines the association with sociodemographic, clinical and lifestyle factors, and differences in European and non-European regions and healthcare systems.

**Methods:**

In this cross-sectional study, 1097 HNC survivors from 11 countries completed the Short-Form Supportive Care Needs Survey (SCNS-SF34) and HNC-specific module (SCNS-HNC), encompassing physical and daily living, psychological, sexuality, HNC-specific and lifestyle domains. Scores were dichotomized per domain and across domains into moderate-high unmet SCNs (yes/no). Logistic regression analyses were used to investigate associated factors.

**Findings:**

Half (50%, proportion 100/200) of HNC survivors had unmet SCNs (overall), especially unmet HNC-specific (40%, 40/100), psychological (25%, 25/100), and physical and daily living (22%, 22/100) needs. Personal (women, lower age), lifestyle (smoking, alcohol consumption) and clinical factors (advanced tumor stage, second primary tumor, multimodality treatment [versus single surgery], poor Karnofsky performance score and comorbidities) were associated with unmet SCNs. Physical and daily living, HNC-specific and overall unmet SCNs were more likely among survivors from Northern Europe compared to Southern and Western Europe. Unmet psychological, sexuality and lifestyle needs were more likely among non-European countries. All unmet SCNs (except psychological) were more likely among survivors with a national health system compared to a social and/or etatist health insurance system.

**Interpretation:**

Half of HNC survivors have unmet SCNs. Insight into healthcare utilization may provide insights how to improve care.

**Funding:**

European Organization for Research and Treatment of Cancer (EORTC) Quality of Life Group.

**Research in context**

**Evidence before this study**

PubMed was searched up to February 2025 using a combination of MeSH terms and keywords for supportive care needs (SCNs) (e.g., supportive care, aftercare) and head and neck cancer (HNC) (e.g., head and neck tumors and neoplasms), namely (*“supportive care needs"[All Fields] OR “after care"[All Fields] OR “need for supportive care"[All Fields] OR “unmet needs"[All Fields] OR “supportive care needs survey"[All Fields] OR “SCNS-SF34"[All Fields]) AND (“head and neck neoplasms"[MeSH Terms] OR (“head"[All Fields] AND “neck"[All Fields] AND “neoplasms"[All Fields]) OR “head and neck neoplasms"[All Fields] OR (“head"[All Fields] AND “neck"[All Fields] AND “cancer"[All Fields]) OR “head and neck cancer"[All Fields])*. In addition, the reference list of eligible studies was examined. Studies which quantitatively investigated SCNs of HNC patients or of mixed cancer patients with HNC as subgroup were included.

**Added value of this study**

Most of the identified studies were cross-sectional studies that focused on HNC patients with a heterogeneous follow-up time or focused on SCNs up to 2 years follow-up only. Limited studies have been published on SCNs of long-term HNC survivors. Also, no study investigated differences among regions or healthcare systems.

**Implications of all the available evidence**

This study showed that HNC survivors also experience unmet SCNs at long-term (>5 years) follow-up. Personal, lifestyle and clinical factors, as well as geographical region and type of healthcare system were found to be associated with unmet SCNs. Further research should focus on differences in actual healthcare utilization among European and non-European regions and healthcare systems, as this may provide handles on how to improve care for HNC survivors.

## Introduction

Every year, over 160,000 people are diagnosed with head and neck cancer (HNC) in Europe.^1^ HNC patients often experience general and HNC-specific symptoms such as pain, fatigue, symptoms of anxiety or depression, and problems with swallowing, speaking, and eating, influencing their health-related quality of life (HRQoL).^2^, ^3^, ^4^ To alleviate these symptoms and to improve HRQoL, supportive care should be provided to the patient, which entails the provision of information and care for daily living, physical and emotional functioning, sexuality and lifestyle.^5^^,^^6^ A systematic review conducted in 2021 identified 15 studies investigating supportive care needs (SCNs) among patients with HNC. This review reported that SCNs were highest in the HNC-specific domain (e.g., problems with chewing or swallowing), followed by a domain on information and health system needs (e.g., information on test results as soon as feasible) and a domain on psychological needs (e.g., fears about the cancer spreading).^7^ Since 2021, several other studies have been conducted.^8^, ^9^, ^10^, ^11^, ^12^ A study on longitudinal changes in SCNs from HNC diagnosis up to 2 years after treatment showed that SCNs diminish over time. At the time of HNC diagnosis, 65% of HNC patients had unmet SCNs (encompassing SCNs for which patients did not receive sufficient help), while 43% had unmet SCNs 2 years after treatment.^11^

Most of the previous studies combined patients with a heterogeneous follow-up time in cross-sectional analyses or focused on (unmet) SCNs up to 2 years follow-up.^7^, ^8^, ^9^, ^10^, ^11^, ^12^ Limited attention has been paid to the (unmet) SCNs of long-term HNC survivors, despite the fact that the 5-year relative survival of HNC patients diagnosed between 2000 and 2007 was about 40%.^13^ Large differences were seen among tumor locations, with 5-year relative survival of 25% (hypopharynx), 39% (oropharynx), 45% (oral cavity), and 59% (larynx).^13^ So far, only two studies have investigated (unmet) SCNs among long-term HNC survivors.^14^^,^^15^ Oskam et al.^14^ reported that there was a high need for a physical therapy (23%) and dental hygiene (46%) among 26 Dutch HNC survivors 8–11 years after treatment. O'Brien et al.^15^ found among 291 Irish HNC survivors >5 years after diagnosis that the highest prevalence of unmet SCNs were reported on the physical and daily living domain (30%), followed by the psychological (27%), health system information (16%), care and support (13%) and sexuality (12%) domain.

To date, no study has investigated differences in (unmet) SCNs among groups of long-term HNC survivors. Previous studies with a short or heterogeneous follow-up showed higher (unmet) SCNs among women, younger patients, those higher educated, those living alone, those diagnosed at a more advanced tumor stage or those with a poorer performance on one or multiple SCNs domains.^7^, ^8^, ^9^, ^10^, ^11^, ^12^ However, other studies found no such difference. Tumor location, tumor treatment, smoking, and alcohol consumption were quite consistently found not to be associated with (unmet) SCNs.^7^, ^8^, ^9^, ^10^, ^11^, ^12^ Environmental factors, including cultural differences among countries or differences in reimbursement of care, have so far not been studied.

Greater understanding of unmet SCNs among long-term HNC survivors, as well as those at risk of experiencing these unmet SCNs, is crucial for improving supportive care for HNC survivors. Therefore, this study aimed to explore unmet SCNs among HNC survivors beyond 5 years after diagnosis, to examine its association with sociodemographic, clinical and lifestyle factors, as well as to identify differences among European and non-European regions and healthcare systems.

## Methods

The European Organization for Research and Treatment of Cancer (EORTC) 1629 study is a cross-sectional, multinational study among HNC survivors.^3^^,^^4^ HNC survivors from 26 medical centers in various countries were invited to participate between October 2018 and October 2021. Inclusion criteria were being diagnosed with cancer of the oral cavity, oropharynx, hypopharynx, nasopharynx, larynx, lip, salivary glands, nasal cavity, paranasal sinuses or being diagnosed with a lymph node carcinoma metastasis of the neck of unknown origin; diagnosis >5 years ago, ability to understand and complete the study, and being willing to participate. Eligible survivors were invited by their treating healthcare professional or a study coordinator via telephone, postal mail or at time of follow-up visit to the medical center. Further information on the study design as well as the sample size calculation can be found in previous publications.^3^^,^^4^

### Supportive care needs

SCNs were measured using the 34-item Short-Form Supportive Care Needs Survey (SCNS-SF34)^6^ and its HNC-specific module (SCNS-HNC).^16^ The SCNS-SF34 consists of 34 items on five underlying domains: physical and daily living needs, psychological needs, sexuality needs, patient care and support needs and health system and information needs.^6^ The SCNS-SF34 domains on patient care and support and health system and information needs were omitted from the survey as the study group deemed them less important at long-term follow-up and to limit the burden of participants, leaving 18 items on the first 3 domains (physical and daily living, psychological, sexuality) to be assessed. The SCNS-HNC contains 11 items covering two underlying domains: HNC-specific functioning (8 items) and lifestyle needs (2 items), and one single item on stoma care and/or voice prosthesis care. The SCNS-SF34 and SCNS-HNC items have a 5-point response scale: “1 = not applicable” for issues that are no problem; “2 = satisfied” for issues on which the survivor needs support but the support is already satisfactory fulfilled; and “3 = low unmet need,” “4 = moderate unmet need,” and “5 = high unmet need” for issues on which a survivor reports respectively a low, moderate or high unmet need for supportive care.^6^ The participants were asked to rate SCNs for the past month.

The SCNS-SF34 has previously been shown to be valid and reliable in different languages, including the Dutch, French, German, Italian and Japanese language.^6^^,^^16^, ^17^, ^18^, ^19^, ^20^ The SCNS-HNC has previously been validated among Dutch HNC survivors,^16^ which showed that the SCNS-HNC is valid and has moderate to good test-retest reliability. If a required translation was unavailable for the SCNS-SF34 (Greek, Hebrew, Portuguese, Norwegian and Swedish) and/or SCNS-HNC (all languages except Dutch), the questionnaires were translated according to the EORTC guidelines.^21^ The questionnaires were first forward translated by two people independently from each other and then the translations were combined into one, followed by a backward translation by another person. The internal consistency of the instruments in this study were, except for a moderate score on the lifestyle domain (alpha = 0·57), good to excellent (Cronbach's alpha of 0·87–0·94).

### Sociodemographic and clinical data

Sociodemographic characteristics (education, living situation, smoking and alcohol use) were collected using a study-specific form. Tumor location, TNM stage, tumor recurrence, treatment, Karnofsky performance score and comorbidity were noted on a case report form. Karnofsky performance status score was used to measure the survivors’ level of functioning in daily activity, physical ability, and self-care on a Likert scale from 10 (moribund) to 100 (fully active). The Charlson Comorbidity Index was used to summarize 19 potential comorbidities.^22^ Patients were categorized into no comorbidities, one comorbidity, or more than one comorbidity. The primary HNC diagnosis under investigation in this study was not coded as a comorbidity. New cancer diagnoses in the last 5 years were coded as comorbidity.

### Country and health system data

Countries were categorized into Northern Europe (two sites in Norway and Sweden), Southern Europe (nine sites in Greece, Italy and Portugal), Western Europe (12 sites in Belgium, Germany, the Netherlands) and non-European countries (three sites in Brazil, Israel and Japan). Healthcare systems were coded following Böhm et al.,^23^ who distinguished healthcare systems based on three dimensions: “regulation (i.e., who sets the rules?),” “financing (i.e., who pays for healthcare),” and “provision (i.e., who delivers healthcare services).” Each of these dimensions could be provided by three actors: namely “state (i.e., government, agencies and public institutions),” “societal (i.e., non-governmental collectively organized actors), and “private (i.e., for-profit or non-profit private entities).” In case multiple actors are involved, the most dominant actor was used. For this manuscript we distinguished three types of systems; a National health system (regulation = state, financing = state, provision = state or private) encompassing 11 sites in Norway, Sweden, Italy, Portugal, Brazil; Social health insurance (regulation = societal, financing = societal, provision = private) encompassing seven sites in Germany; and etatist health insurance (regulation = state, financing = societal, provision = private) encompassing eight sites in Belgium, the Netherlands, Israel, Japan and Greece.

### Statistical analyses

Baseline characteristics of the study population were described using frequencies and percentages or means and standard deviations (SD). SCNs domain scores were converted to a 0–100 score, with a higher score indicating a higher level of SCNs. In addition, scores were dichotomized into having at least one moderate to high unmet SCN (score of 4 or 5) per domain as well as at least one moderate to high unmet SCNs across all domains (called overall unmet SCN). In case of missing data in less than 50% of the SCNS-SF34 or SCNS-HNC items of a particular domain, a total score was calculated assuming that the mean score of the missing items was the same as the completed items. To investigate unmet SCNs (yes/no) and its association with sociodemographic, clinical and lifestyle factors, and differences in European and non-European regions and healthcare systems, three series of logistic regression analyses were performed investigating: (A) sociodemographic, clinical and lifestyle factors associated with unmet SCNs; (B) differences among European and non-European regions (when adjusted for factors significant in part A); and (C) differences among healthcare systems (when adjusted for factors significant in part A). Backward logistic regression analysis using a p-value for entry of <0·10 was performed. In case the p-value in the final model was <0·05, the factor was considered statistically significant. The statistical analyses were performed using the IBM Statistical Package for the Social Science (SPSS) version 28 (IBM Corp., Armonk, NY USA).

### Ethics approval

The medical ethical committee of Medical Association of Rhineland-Palatinate (Germany) and, if needed, the ethics committees of participating medical centers approved the study. All participating survivors provided written informed consent.

### Role of the funding source

The study was funded by The European Organization for Research and Treatment of Cancer (EORTC) Quality of Life Group (grant number 1629). The EORTC Quality of Life Group business model involves charges for commercial companies using EORTC instruments. The funder had no role in study design, data collection, data analysis, data interpretation, or writing of the report. The corresponding author had full access to all of the data and the final responsibility to submit for publication.

## Results

In total, 1114 HNC survivors from 26 sites in 11 countries (Belgium, Greece, Germany, Italy, Netherlands, Norway, Portugal and Sweden in Europe and Brazil, Israel and Japan) were included in this study. Of them, 1097 survivors completed the questionnaire on SCNs (98%). The mean age of the study population was 66 ± 10 years (Table 1). The majority was male (71%), had >10 years of education (51%) and lived with someone (78%). Most survivors were diagnosed with an oropharyngeal tumor (34%), with tumor stage IV (40%) and treated with surgery in combination with (chemo)radiotherapy (38%). Median follow-up was 8 years (range 5–36 years). About half (54%) of the HNC survivors were treated in a country with a national healthcare system, 28% had an etatist health insurance system, and 18% a social health insurance system.

**Table 1:** Sociodemographic and clinical characteristics of the HNC survivors.

	HNC survivors N = 1097^a^	
	N, median or mean (SD)	% or [range]
Mean age (SD)	66 (10)	[23–93]
Sex		
Men	776	71
Women	321	29
Education^b^		
<10 years	369	35
10 years	160	15
>10 years	540	51
Living situation^b^		
Living alone	234	22
Living together	844	78
Smoking status^b^		
Never smoker	314	29
Former smoker	625	58
Current smokers	139	13
Alcohol consumption^b^		
Abstainers	273	26
Sometimes	524	51
Almost daily (≥4 times a week)	240	23
Tumor location		
Oral cavity	240	22
Oropharynx	372	34
Hypopharynx	50	5
Nasopharynx	85	8
Larynx	202	18
Salivary glands	60	5
Nasal cavity or paranasal sinus	37	3
Unknown primary	51	5
TNM stage^b^		
Stage I	218	21
Stage II	173	16
Stage III	244	23
Stage IV	426	40
Second primary tumor^b^^,^^c^		
Yes	162	15
No	929	85
Recurrence^b^		
Yes	128	12
No	959	88
Treatment^b^		
Surgery	128	12
Radiotherapy	132	12
Chemoradiotherapy	308	28
(Chemo)radiotherapy and neck dissection	111	10
Surgery and (chemo)radiotherapy	417	38
Years since primary diagnosis (median)	9·0	[5–36]
Karnofsky performance score (10–100 score)^b^	89 (11)	[40–100]
Charlson Comorbidity Index	0·76 (1·4)	[0–10]
No comorbidity	699	64
1 comorbidity	205	19
>1 comorbidity	193	18
Healthcare system		
National health system	588	54
Social health insurance	199	18
Etatist health insurance	310	28
Region		
Northern Europe	249	23
Southern Europe	238	22
Western Europe	462	42
Non-European countries	148	13

a All variables reflect the situation at the time of SCNS completion.

b Data are missing on education level (missing = 28), living situation (missing = 19), smoking status (missing = 19), alcohol consumption (missing = 60), TNM stage (missing = 36), treatment (missing = 1), second primary (missing = 15), recurrence (missing = 10) and Karnofsky performance score (missing = 24).

c Second primary tumor, of which 34% HNC.

### Unmet supportive care needs

In total, 1082 of the 1097 HNC survivors had complete data on all SCN domains. Of these 1082 HNC survivors, 50% (n = 541) reported at least one unmet SCN (called overall unmet need). Unmet SCNs were most often reported on the HNC-specific domain (40%), followed by the psychological (25%), physical and daily living (22%), sexuality (11%), and lifestyle (7%) domain (Table 2). Most prevalent unmet SCNs related to problems with dry mouth and/or sticky mucus (24%), problems with chewing and/or swallowing (20%), problems with the mobility of neck or shoulders (17%), lack of energy/tiredness (12%), and problems with weight (underweight or overweight) (12%).

**Table 2:** Proportion of unmet supportive care needs and mean domain score.

SCNS domains and items^1^	N (%) with moderate-high unmet needs	Mean (SD) per SCNS domain
Physical and daily living needs	239 (22%)	17 (23)
Pain	110 (10%)	
Lack of energy/tiredness	135 (12%)	
Feelings unwell a lot of the time	58 (5%)	
Work around the home	98 (9%)	
Not being able to do the things you used to do	123 (11%)	
Psychological needs	272 (25%)	18 (22)
Anxiety	94 (9%)	
Feeling down or depressed	94 (9%)	
Feelings of sadness	95 (9%)	
Fears about the cancer spreading	123 (11%)	
Worry that the results of treatment are beyond your control	75 (7%)	
Uncertainty about the future	116 (11%)	
Learning to feel in control of your situation	79 (7%)	
Keeping a positive look	77 (7%)	
Feelings about death and dying	75 (7%)	
Concerns about the worries of those close to you	131 (12%)	
Sexuality needs	123 (11%)	14 (24)
Changes in sexual feelings	103 (10%)	
Changes in your sexual relationships	94 (9%)	
To be given information about sexual relationships	51 (5%)	
HNC-specific functioning needs	435 (40%)	22 (21)
Problems with chewing and/or swallowing	215 (20%)	
Problems with dry mouth and/or sticky mucus	257 (24%)	
Problems with weight (underweight or overweight)	132 (12%)	
To be informed on nutrition	76 (7%)	
Difficulty speaking	106 (10%)	
Problems with hearing	125 (11%)	
Oral hygiene	63 (6%)	
Problems with mobility of neck or shoulders	184 (17%)	
Lifestyle needs	73 (7%)	8 (17)
Quit smoking	58 (5%)	
Quit drinking	28 (3%)	
Single item		
Care of your stoma and/or voice prosthesis	36 (3%)	
≥1 moderate/high unmet need	541 (50%)	

All items were addressed using the following question: “In the last month what was your level of help with [item].” All items were answered on a 5-point response scale: “1 = not applicable” for issues that are no problem; “2 = satisfied” for issues on which the survivor needs support but the support is already satisfactory fulfilled; and “3 = low unmet need”; “4 = moderate unmet need”; and “5 = high unmet need” for issues on which a survivor reports respectively a low, moderate or high unmet need for supportive care. Scores were dichotomized into having (yes/no) moderate to high unmet supportive care needs (score of 4 or 5). Abbreviations: SCNS, Supportive Care Needs Survey; N, number, SD, standard deviation; HNC, head and neck cancer.

### Unmet supportive care needs in relation to sociodemographic, clinical and lifestyle factors

**Fig. 1:**
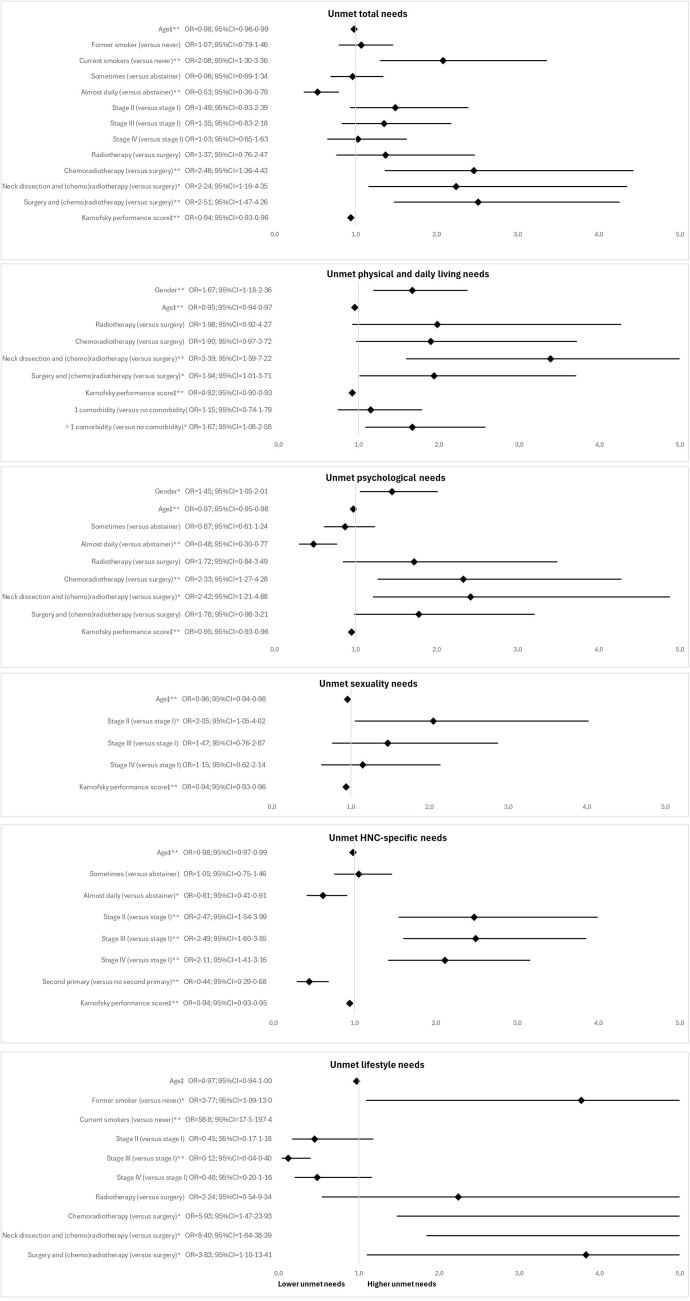
**Forrest plots of sociodemographic, clinical and lifestyle factors associated with unmet SCNs per SCNS domain in the final multivariable logistic regression analyses.** Abbreviations: SCN, supportive care needs; SCNS, Supportive Care Needs Survey; RT, radiotherapy; OR, odds ratio; 95% CI, 95% confidence interval. An ∗ indicates a p-value <0·05 and ∗∗ indicates a p-value <0·01. ‡ Per one point increase in either age or Karnofsky performance score.

Multivariable analyses on factors associated with unmet SCNs are presented in Fig. 1 (for univariable analyses see Appendix S1). Overall unmet SCNs were associated with lower age, being a current smoker (versus no history of smoking), no alcohol consumption (versus almost daily consumption), being treated with chemoradiotherapy, neck dissection combined with (chemo)radiotherapy, or surgery combined with (chemo)radiotherapy (versus surgery only) and a poorer Karnofsky performance score.

Regarding the SCNS-SF34 domains, unmet physical and daily living needs were associated with being a woman, lower age, being treated with neck dissection combined with (chemo)radiotherapy or surgery combined with (chemo)radiotherapy (versus surgery only), a poorer Karnofsky performance score and having more than one comorbidity. Unmet psychological needs were found to be associated with being a woman, lower age, no alcohol consumption (versus almost daily consumption), those treated with chemoradiotherapy or neck dissection combined with (chemo) radiotherapy (versus surgery only) and those with a poorer Karnofsky performance score. Unmet sexuality needs were found to be associated with lower age, TNM stage II (versus stage I) and a poorer Karnofsky performance score.

Concerning the SCNS-HNC domains, unmet HNC-specific needs were found to be associated with lower age, no alcohol consumption (versus almost daily consumption), a more advanced TNM stage, not being diagnosed with a second primary malignancy and a poorer Karnofsky performance score. Unmet lifestyle needs were found to be associated with being a former or current smoker (versus no smoker), TNM stage I (versus stage III) and being treated with chemoradiotherapy, a neck dissection combined with (chemo) radiotherapy or surgery combined with (chemo)radiotherapy (versus surgery alone).

### Unmet supportive care needs among European and non-European regions and health systems

**Fig. 2:**
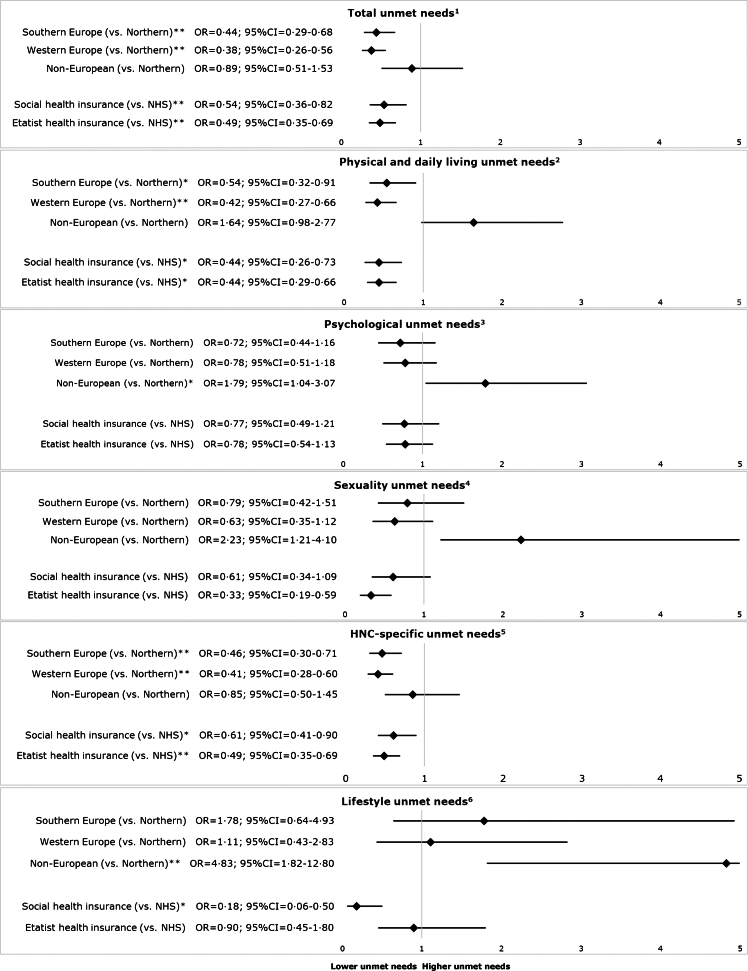
**Forrest plots of the association of region and healthcare system with unmet SCNs per SCNS domain in multivariable logistic regression analyses.** Abbreviations: SCN, supportive care needs; SCNS, Supportive Care Needs Survey; NHS, National Health System; OR, odds ratio; 95% CI, 95% confidence interval. 1, adjusted for age, smoking, alcohol, TNM stage, treatment and Karnofsky performance score; 2, adjusted for sex, age, treatment, Karnofsky performance score and comorbidity; 3, adjusted for sex, age, alcohol, treatment, Karnofsky performance score; 4, adjusted for age, TNM stage and Karnofsky performance score; 5, adjusted for age, alcohol, TNM stage, second primary tumor and Karnofsky performance score; 6, adjusted for age, smoking, TNM stage and treatment. An ∗ indicates a p-value <0·05 and ∗∗ indicates a p-value <0·01.

Adjusted analyses showed that overall unmet SCNs and unmet physical and daily living and HNC-specific needs were more likely among HNC survivors from Northern Europe than among HNC survivors from Southern Europe and Western Europe. Unmet psychological, sexuality and lifestyle needs were more likely among non-European countries (versus Northern Europe) (Fig. 2 and Appendix S2).

When comparing healthcare systems, it was found that unmet overall, physical and daily living, and HNC-specific needs were more likely among HNC survivors with a national health system compared to both a social health insurance and etatist health insurance system. Unmet sexuality and lifestyle needs were also more likely among HNC survivors with a national health system compared to HNC survivors with respectively an etatist health insurance or social health insurance system.

## Discussion

This study showed that 50% of long-term HNC survivors had unmet SCNs. Personal (gender, age), lifestyle (smoking, alcohol consumption) and clinical factors (tumor stage, second primary tumor, multimodality treatment, Karnofsky performance score, and comorbidities), geographical region (Northern Europe and non-European countries) and type of healthcare system (national health system) were associated with unmet SCNs.

Our finding that 50% of HNC survivors experience unmet SCNs (overall) is difficult to compare to previous studies,^7^, ^8^, ^9^, ^10^, ^11^, ^12^ as studies often differ with regard to the measurement instrument used (SCNS-SF34 or other), the number of SCNs taken into account (all SCNS-SF34 and HNC-specific domains or a subset), and the used definition for unmet SCNs (a focus on unmet *moderate-high* SCNs or unmet *low-high* SCNs). Nonetheless, our findings on unmet *moderate-high* SCNs (psychological (25%), physical and daily living (22%), sexuality (11%)) are somewhat comparable to the unmet *low-high* SCNs reported by O'Brien et al.^15^ who found among HNC survivors >5 years after diagnosis unmet *low-high* SCNs of 30% on the physical and daily living domain, 27% on the psychological domain and 12% on the sexuality domain. Our results are higher compared to a study among HNC survivors 2 years after the end of treatment^11^ (unmet *moderate-high* SCNs of 18% on the HNC-specific domain, 17% on the physical and daily living domain, 14% on the psychological domain, 8% on the sexuality domain and 5% on the lifestyle domain) and a study (included in the systematic review) among HNC survivors immediately after treatment^24^ (unmet *moderate-high* needs of 19% (physical and daily living) and 8% (sexuality)). Overall, it seems that unmet SCNs in our study were higher than those reported in previous studies. This might be related to changes in HNC treatment and/or supportive care in the last decades.^25^ In our study, survivors were diagnosed with HNC between 1985 and 2017 (74% before 2014, 24% before 2010), whereas survivors in the study of O'Brien et al.^15^ were diagnosed before April 2012 (no lower limit reported), survivors in the study of Henry et al.^24^ were diagnosed between 2012 and 2015, and survivors in the study of Molenaar et al.^11^ were diagnosed between 2014 and 2018. An alternative explanation may be that, due to the long follow-up, HNC survivors of our study were older compared to the previous studies.

Remarkably, cancer treatment was found to be associated with unmet SCNs in our study, whereas, except for one study,^15^ previous studies found no such association.^7^, ^8^, ^9^, ^10^, ^11^, ^12^ This might again be explained by changes in tumor treatment/supportive care in the last decade. Another explanation may be that cancer treatment causes late effects which may impact SCNs at long-term follow-up. This may also explain why the study of O'Brien et al.^26^ in which 50% had a follow-up of over 5 years also showed significant associations between cancer treatment and SCNs.

Other factors which were found to be associated with unmet SCNs were generally in line with previous research, namely higher unmet SCNs among women, younger survivors, those with a more advanced tumor stage, or a poorer performance status.^7^, ^8^, ^9^, ^10^, ^11^, ^12^ A difference between our findings and previous findings is that we found higher unmet needs among survivors who currently smoke (versus those with no history of smoking), whereas previous studies found no such difference.^7^, ^8^, ^9^, ^10^, ^11^, ^12^ The reason for this discrepancy might be the large sample size of our study (n = 1097) which enabled us to differentiate between well-sized subgroups of never, former and current smokers (the smallest subgroup contained n = 139 survivors). Remarkably, also no alcohol consumption (versus frequent alcohol consumption) was associated with SCNs. An explanation may be that we did not have data on history of alcohol consumption; therefore, the non-drinking group contained both patients with and without a history of excessive alcohol consumption. Another remarkable finding was that being diagnosed with a second primary tumor was, in contrast to previous studies who found no association or *higher* (unmet) SCNs,^7^, ^8^, ^9^, ^10^, ^11^, ^12^ associated with *lower* HNC-specific needs in our study. A potential explanation may be that these survivors already adapted to the situation and showed a response shift. Also, it may be that these survivors are more regularly seen by healthcare professionals and therefore are more likely to receive supportive care. Another explanation may be that two thirds of the second primaries were non-HNC tumors, which may have resulted in HNC survivors shifting their attention to non-HNC needs.

Besides the above-mentioned factors, unmet SCNs differed among European and non-European regions and healthcare systems. It was found that overall SCNs, physical and daily living and HNC-specific unmet needs were more likely among Northern Europe than among Southern Europe and Western Europe, whereas psychological, sexuality and lifestyle needs were more likely among non-European countries (versus Northern Europe). In addition, unmet SCNs were more likely among HNC survivors with a national health system compared to those with a social health insurance or etatist health insurance system. A potential explanation for the differences found among regions and healthcare systems might relate to the fact that we focused on patient's self-perceived unmet SCNs and not on objective differences in actual received care. European and non-European regions and healthcare systems have different cultures. In some cultures, the informal social environment of survivors may provide high levels of support, whereas in other cultures patients may place high demands and high expectations on formal healthcare, which might influence their evaluation of unmet SCNs.^27^ Cultural differences might also explain differences in unmet psychological and sexuality needs, as in some countries (or cultures) it may be more common to talk about psychological or sexual issues than in others.^28^^,^^29^ We, unfortunately, did not collect information on race, ethnicity or religion. Another potential explanation for the higher prevalence of unmet SCNs in Northern Europe may be that Northern Europe is less densely populated and therefore may, in general, have larger travel distances to supportive care. This is in line with a recent systematic review which showed specific SCNs among HNC patients living in rural areas, including needs with regard to traveling, healthcare access and financial problems.^30^ Further insight is needed into actual healthcare utilization among European and non-European regions and healthcare systems, as this will enable comparison of findings on SCNs and actual care use, and thereby may provide handles on how to improve and further tailor care for HNC survivors.

Strengths of this study were the large sample size of >1000 HNC survivors and the international collaboration. A limitation is that we do not have data on survivors who declined participation and are therefore unable to draw conclusions on generalizability of our findings. Another potential limitation is that in some countries survivors from multiple centers participated, whereas in other countries only one center participated. Results may therefore not be representative to all centers in the same region and/or healthcare system. Also, all participating countries were categorized regarding their main type of healthcare system. It should, however, be noted that some countries (e.g., Brazil, Germany, Greece) have both national and private health insurance. In addition, in some countries, national health insurance may be in place, but this is not always sufficient to encounter the needs of the entire population. Another potential limitation is that we used the SCNS-SF34 and SCNS-HNC. The SCNS-SF34 and to a lesser extent the SCNS-HNC have been translated and validated in several countries. For this study, however, we had to make several additional translations. Although these translations followed the EORTC translation guideline (40) and the SCNS-SF34 and SCNS-HNC showed, except for the lifestyle domain, good to excellent internal consistency, further research is warranted on the validity of these new translations. Moreover, a limitation of this study is that we do not know whether the identified unmet SCNs are the direct or indirect result of HNC and its treatment or the consequence of natural aging. Finally, some statistical limitations need to be taken into account. SCN data were dichotomized into unmet needs yes/no and thereby reduced the statistical power; and a stepwise regression analysis was used which may result in overfitting of the data. We tried to limit this problem by including only variables for which we hypothesized a relevant relationship. Other statistical limitations are that we did not conduct multilevel analyses, whereas HNC survivors treated in the same center are not entirely independent (e.g., the routine follow-up protocol and the received care are likely comparable).

In conclusion, we found that 50% of long-term HNC survivors had unmet SCNs especially on the HNC-specific domain. Personal, lifestyle and clinical factors, as well as geographical region and type of healthcare system, were found to be associated with unmet SCNs. Further research is needed into whether current innovations in treatment and supportive care have improved unmet SCNs, as this study population was treated quite some time ago (in between 1985 and 2017). Also, further insight is needed into actual healthcare utilization among European and non-European regions and healthcare systems, as this will provide handles on how to improve care for HNC survivors.

## Contributors

FJ: Conceptualization, data curation, formal analysis, investigation, methodology, resources, visualization, writing—original draft; SEJE: investigation, resources, writing—original draft; KJT: Conceptualization, data curation, investigation, project administration, resources, validation, writing—review & editing; CDA: Conceptualization, investigation, resources, writing—review & editing; KB: Conceptualization, investigation, resources, writing—review & editing; GLA Investigation, resources, writing—review & editing; BBH: Conceptualization, investigation, resources, writing—review & editing; FD: Conceptualization, investigation, resources, writing—review & editing; RRG Conceptualization, investigation, resources, writing—review & editing; AAJ: Conceptualization, investigation, resources, writing—review & editing; EH: Conceptualization, investigation, resources, writing—review & editing; MS: Investigation, resources, writing—review & editing; GF: Investigation, resources, writing—review & editing; OGL: Conceptualization, investigation, resources, writing—review & editing; JI: Conceptualization, investigation, resources, writing—review & editing; TD: Investigation, resources, writing—review & editing; AF: Investigation, resources, writing—review & editing; AnB: Conceptualization, investigation, resources, writing—review & editing; UW: Conceptualization, investigation, resources, writing—review & editing; NK: Conceptualization, investigation, resources, writing—review & editing; MK: Conceptualization, investigation, resources, writing—review & editing; PB: Conceptualization, investigation, resources, writing—review & editing; MoP: Conceptualization, investigation, resources, writing—review & editing; SN: Investigation, resources, writing—review & editing; JCS: Conceptualization, investigation, resources, writing—review & editing; CS: Investigation, resources, writing—review & editing; PS: Investigation, resources, writing—review & editing; FT. Investigation, resources, writing—review & editing; AyB: Investigation, resources, writing—review & editing; PP: Investigation, resources, writing—review & editing; MiP: Investigation, resources, writing—review & editing; AP Investigation, resources, writing—review & editing; NS: Investigation, resources, writing—review & editing; AK: Investigation, resources, writing—review & editing; SS: Conceptualization, funding acquisition, investigation, project administration, resources, supervision, writing—review & editing; IMVL: Conceptualization, funding acquisition, investigation, resources, supervision, writing—original draft.

## Data sharing statement

Data can be requested from the data repository of the EORTC (https://www.eortc.org/data-sharing/).

## Declaration of interests

FJ reported to have received a research grant from the Maarten van der Weijden Foundation (payment to institution). KJT reported to have received a research grant for the European Organisation for Research and Treatment of Cancer (EORTC) (payment to institution), payment of a monthly honoraria for work supporting the EORTC Quality of Life Group Executive Committee with administrative aspects of Quality of Life Group's project, and support for attending meetings, i.e., the EORTC Quality of Life Group pays the hotel and travel costs to attend the EORTC Quality of Life Group Meetings and Head and Neck Group meetings. OGL reported to be advisor of the German Society of Otorhinolaryngology, Head and Neck Surgery (unpaid). NK reported to have received research grants from ONO PHARMACEUTICAL, Bristol-Meyers Squibb, GSK, Adlai Nortye, Boehringer Ingelheim, Abbvie and AstraZeneca Co., Ltd. (all paid to institution), and to have received honoraria for lectures from MSD, Ono Pharmaceutical, Bristol Meyers Squibb, AstraZeneca Co. and Merck Biopharma. MoP reported to have received payment from BD Switzerland S.r.l. and Hinovia S.r.l. (payment to institution). SS reported to have received a research grant from the EORTC (payment to institute), and to have received payment from Lilly for reviewing papers for their quality of life award, outside of this study. All other authors report no conflict of interest.
