# Supplementary material for: Unmet supportive care needs among head and neck cancer survivors beyond 5 years after diagnosis: a multinational cohort study
Source: Lancet Reg Health Eur. 2025 Oct 16;59:101495. doi: 10.1016/j.lanepe.2025.101495 (PMC12553064; doi:10.1016/j.lanepe.2025.101495)
Supplement: Supplementary Table 1 [file mmc1.docx]

**Appendix 1. Univariable logistic regression analyses on factors associated with SCNs per SCNS domain**

|  | **Total** | **SCNS-SF34** |  |  | **SCNS-HNC^2^** |  |
| --- | --- | --- | --- | --- | --- | --- |
|  |  | **Physical & daily living needs** | **Psychological needs** | **Sexuality needs** | **HNC-specific needs** | **Lifestyle needs** |
|  | OR [95%CI] | OR [95%CI] | OR [95%CI] | OR [95%CI] | OR [95%CI] | OR [95%CI] |
| **Sex** |  |  |  |  |  |  |
| - Men | REF | REF | REF | REF | REF | REF |
| - Women | 1·32 [1·02-1·72] | 1·62 [1·19-2·19]* | 1·50 [1·12-2·00]* | 0·96 [0·64-1·46] | 1·41 [1·08-1·83]* | 0·36 [0·18-0·72]* |
| **Age** | 0·98 [0·96-0·99]* | 0·97 [0·96-0·99]* | 0·97 [0·96-0·99]* | 0·97 [0·95-0·99]* | 0·98 [0·97-0·99]* | 0·96 [0·94-0·98]* |
| **Education** |  |  |  |  |  |  |
| - < 10 years | REF | REF | REF | REF | REF | REF |
| - 10 years | 0·78 [0·53-1·13] | 0·82 [0·53-1·29] | 0·61 [0·40-0·95]* | 0·60 [0·33-1·10] | 0·71 [0·48-1·04] | 0·87 [0·45-1·70] |
| - > 10 years | 0·78 [0·60-1·02] | 0·74 [0·54-1·02] | 0·65 [0·48-0·87]* | 0·60 [0·40-0·90]* | 0·78 [0·59-1·02] | 0·48 [0·28-0·82]* |
| **Living situation** |  |  |  |  |  |  |
| - Living alone | REF | REF | REF | REF | REF | REF |
| - Living together | 1·05 [0·79-1·41] | 1·01 [0·71-1·44] | 0·91 [0·66-1·27] | 1·33 [0·81-2·18] | 1·05 [0·78-1·42] | 0·85 [0·48-1·49] |
| **Smoking status** |  |  |  |  |  |  |
| - Never smoker | REF | REF | REF | REF | REF | REF |
| - Former smoker | 1·04 [0·79-1·37] | 0·95 [0·68-1·33] | 0·84 [0·61-1·15] | 1·05 [0·67-1·64] | 1·16 [0·87-1·54] | 3·42 [1·01-11·59]* |
| - Current smokers | 2·48 [1·62-3·78] | 1·56 [0·99-2·46] | 1·71 [1·11-2·63]* | 1·55 [0·86-2·80] | 1·87 [1·25-2·81]* | 55·7 [17·0-183·0]* |
| **Alcohol consumption** |  |  |  |  |  |  |
| - Never | REF | REF | REF | REF | REF | REF |
| - Sometimes | 0·73 [0·54-0·98]* | 0·63 [0·45-0·89]* | 0·73 [0·53-1·01] | 0·69 [0·44-1·09] | 0·81 [0·60-1·09] | 0·77 [0·41-1·42] |
| - Almost daily | 0·46 [0·32-0·65]* | 0·55 [0·36-0·85]* | 0·39 [0·26-0·61]* | 0·62 [0·35-1·09] | 0·50 [0·35-0·73]* | 1·20 [0·62-2·35] |
| **Tumor location** |  |  |  |  |  |  |
| - Oral cavity | REF | REF | REF | REF | REF | REF |
| - Oropharynx | 0·70 [0·51-0·98]* | 0·82 [0·56-1·21] | 0·88 [0·60-1·27] | 1·03 [0·60-1·77] | 0·76 [0·55-1·06] | 0·56 [0·29-1·08] |
| - Hypopharynx | 0·81 [0·44-1·49] | 0·61 [0·27-1·38] | 1·20 [0·62-2·35] | 1·42 [0·58-3·52] | 0·50 [0·26-0·97]* | 1·77 [0·70-4·44] |
| - Nasopharynx | 1·26 [0·76-2·10] | 1·31 [0·75-2·27] | 1·17 [0·68-2·03] | 1·75 [0·86-3·57] | 1·45 [0·88-2·39] | 0·69 [0·25-1·89] |
| - Larynx | 0·58 [0·40-0·85]* | 0·83 [0·53-1·31] | 0·77 [0·49-1·20] | 1·20 [0·66-2·19] | 0·55 [0·37-0·81]* | 0·88 [0·44-1·78] |
| - Salivary glands / parotid | 0·80 [0·45-1·42] | 0·89 [0·45-1·76] | 0·86 [0·44-1·66] | 1·37 [0·58-3·23] | 0·68 [0·38-1·22] | 0·57 [0·16-1·99] |
| - Nasal cavity or sinus | 0·73 [0·37-1·47] | 1·16 [0·53-2·54] | 1·92 [0·94-3·92] | 1·06 [0·35-3·25] | 0·56 [0·27-1·17] | 0 [0-0] |
| - Unknown primary | 0·64 [0·35-1·17] | 0·42 [0·17-1·03] | 0·60 [0·28-1·31] | 0·74 [0·25-2·25] | 0·70 [0·37-1·30] | 1·18 [0·42-3·30] |
| **TNM stage** |  |  |  |  |  |  |
| - Stage I | REF | REF | REF | REF | REF | REF |
| - Stage II | 1·98 [1·32-2·99]* | 2·01 [1·23-3·29]* | 1·99 [1·24-3·20]* | 2·52 [1·34-4·75]* | 2·46 [1·60-3·76]* | 0·96 [0·44-2·09] |
| - Stage III | 1·73 [1·20-2·51]* | 1·38 [0·86-2·22] | 1·72 [1·10-2·69]* | 1·44 [·76-2·73] | 2·08 [1·40-3·09]* | 0·32 [0·12-0·84]* |
| - Stage IV | 1·73 [1·24-2·42]* | 1·58 [1·03-2·43]* | 1·53 [1·01-2·30]* | 1·43 [·80-2·57] | 2·12 [1·48-3·03]* | 1·17 [0·63-2·16] |
| **Second primary tumor** |  |  |  |  |  |  |
| - No | REF | REF | REF | REF | REF | REF |
| - Yes | 1·08 [0·77-1·52] | 1·30 [0·89-1·92] | 1·15 [0·79-1·68] | 1·41 [0·87-2·31] | 0·70 [0·49-1·00)* | 0·92 [0·46-1·84] |
| **Recurrence** |  |  |  |  |  |  |
| - No | REF | REF | REF | REF | REF | REF |
| - Yes | 1·41 [0·97-2·06] | 1·33 [0·87-2·03] | 1·54 [1·03-2·29]* | 1·23 [0·71-2·13] | 1·26 [0·87-1·83] | 0·81 [0·36-1·81] |
| **Treatment** |  |  |  |  |  |  |
| - Surgery | REF | REF | REF | REF | REF | REF |
| - Radiotherapy | 1·24 [0·74-2·07] | 1·76 [0·88-3·52] | 1·41 [0·73-2·74] | 0·77 [0·31-1·93] | 1·58 [0·91-2·74] | 1·72 [0·49-6·03] |
| - Chemoradiotherapy | 2·38 [1·54-3·68]* | 2·08 [1·14-3·81]* | 2·41 [1·38-4·20]* | 1·60 [0·79-3·22] | 2·62 [1·63-4·20]* | 2·51 [0·85-7·41] |
| - Neck dissection and (chemo)radiotherapy | 1·96 [1·16-3·33]* | 2·89 [1·47-5·71]* | 2·24 [1·17-4·30]* | 1·93 [0·86-4·33] | 2·30 [1·31-4·04]* | 2·71 [0·81-9·07] |
| - Surgery and (chemo)radiotherapy | 2·79 [1·83-4·25}* | 2·40 [1·34-4·31]* | 2·23 [1·30-3·84]* | 1·34 [0·67-2·66] | 2·72 [1·72-4·29]* | 2·42 [0·84-7·01] |
| **Years since primary** **diagnosis** | 0·99 [0·96-1·02] | 1·01 [0·98-1·05] | 1·00 [0·97-1·04] | 1·02 [0·97-1·06] | 1·01 [0·98-1·04] | 0·98 [0·91-1·05] |
| **Karnofsky performance score (10-100 score)** | 0·94 [0·93-0·96]* | 0·92 [0·91-0·93]* | 0·95 [0·94-0·96]* | 0·95 [0·94-0·96]* | 0·95 [0·93-0·96]* | 0·96 [0·94-0·98]* |
| **Charlson Comorbidity Index** |  |  |  |  |  |  |
| - No comorbidity | REF | REF | REF | REF | REF | REF |
| - 1 comorbidity | 1·06 [0·78-1·46] | 1·11 [0·75-1·64] | 0·86 [0·59-1·25] | 0·97 [0·58-1·62] | 1·20 [0·87-1·64] | 0·81 [0·41-1·59] |
| - > 1 comorbidity | 1·42 [1·03-1·97}* | 2·28 [1·60-3·24]* | 1·26 [0·89-1·80] | 1·45 [0·91-2·32] | 1·30 [0·94-1·80] | 1·30 [0·72-2·34] |

Abbreviations: SCN, supportive care needs; SCNS, Supportive Care Needs Survey; SCNS-SF34, 34-item Short-Form Supportive Care Needs Survey; SCNS-HNC, HNC-specific Supportive Care Needs Survey; OR, odds ratio; 95%CI, 95% confidence interval; REF, reference

An * indicates a p-value < 0·05
