# Supplementary material for: Unmet supportive care needs among head and neck cancer survivors beyond 5 years after diagnosis: a multinational cohort study
Source: Lancet Reg Health Eur. 2025 Oct 16;59:101495. doi: 10.1016/j.lanepe.2025.101495 (PMC12553064; doi:10.1016/j.lanepe.2025.101495)
Supplement: Supplementary Table 2 [file mmc2.docx]

**Appendix 2. Univariable and multivariable logistic regression analyses on SCNs among European and non-European regions and type of healthcare system per SCNS domain**

|  | **Total** |  | **SCNS-SF34** |  |  |  |  |  | **SCNS-HNC** |  |  |  |
| --- | --- | --- | --- | --- | --- | --- | --- | --- | --- | --- | --- | --- |
|  |  |  | **Physical & daily living needs** | | **Psychological needs** | | **Sexuality needs** | | **HNC-specific functioning needs** | | **Lifestyle needs** | |
|  | OR  [95%CI] | Adj. OR^1^ [95%CI] | OR  [95%CI] | Adj. OR^2^ [95%CI] | OR  [95%CI] | Adj. OR^3^ [95%CI] | OR  [95%CI] | Adj. OR^4^ [95%CI] | OR  [95%CI] | Adj. OR^5^ [95%CI] | OR  [95%CI] | Adj. OR^6^ [95%CI] |
| **Region** |  |  |  |  |  |  |  |  |  |  |  |  |
| Northern Europe | REF | REF | REF | REF | REF | REF | REF | REF | REF | REF | REF | REF |
| Southern Europe | 0·56  [0·39-0·81]** | 0·44  [0·29-0·68]** | 0·64  [0·41-1·00] | 0·54  [0·32-0·91]* | 0·78  [0·51-1·20] | 0·72  [0·44-1·16] | 0·91  [0·50-1·66] | 0·79  [0·42-1·51] | 0·51  [0·35-0·74]** | 0·46  [0·30-0·71]** | 2·63  [1·23-6·12]* | 1·78  [0·64-4·93] |
| Western Europe | 0·56  [0·41-0·76]** | 0·38  [0·26-0·56]** | 0·65  [0·44-0·94]* | 0·42  [0·27-0·66]** | 0·85  [0·59-1·22] | 0·78  [0·51-1·18] | 0·84  [0·49-1·42] | 0·63  [0·35-1·12] | 0·56  [0·41-0·77]** | 0·41  [0·28-0·60]** | 1·68  [0·74-3·80] | 1·11  [0·43-2·83] |
| Non-European countries | 1·82  [1·18-2·82]** | 0·89  [0·51-1·53] | 2·21  [1·43-3·42]** | 1·64  [0·98-2·77] | 2·38  [1·54-3·67]** | 1·79  [1·04-3·07]* | 2·99  [1·71-5·21]** | 2·23  [1·21-4·10]** | 1·46  [0·97-2·20] | 0·85  [0·50-1·45] | 5·30  [2·29-12·3]** | 4·83  [1·82-12·8]** |
| **Healthcare system** |  |  |  |  |  |  |  |  |  |  |  |  |
| National health system | REF | REF | REF | REF | REF | REF | REF | REF | REF | REF | REF | REF |
| Social health insurance | 0·65  [0·47-0·90]** | 0·54  [0·36-0·82]** | 0·55  {0·36-0·85]** | 0·44  [0·26-0·73]* | 0·68  [0·46-1·01] | 0·77  [0·49-1·21] | 0·71  [0·43-1·20] | 0·61  [0·34-1·09] | 0·68  [0·49-0·95]* | 0·61  [0·41-0·90]* | 0·37  [0·16-0·88]* | 0·18  [0·06-0·50]* |
| Etatist health insurance | 0·57  [0·43-0·76]** | 0·49  [0·35-0·69]** | 0·69  [0·49-0·97]* | 0·44  [0·29-0·66}* | 0·81  [0·59-1·11] | 0·78  [0·54-1·13] | 0·45  [0·27-0·74]** | 0·33  [0·19-0·59]** | 0·59  [0·44-0·79]** | 0·49  [0·35-0·69]** | 0·79  [0·46-1·37] | 0·90  [0·45-1·80] |

Abbreviations: SCN, supportive care needs; SCNS, Supportive Care Needs Survey; SCNS-SF34, 34-item Short-Form Supportive Care Needs Survey; SCNS-HNC, HNC-specific Supportive Care Needs Survey; OR, odds ratio; 95%CI, 95% confidence interval; REF, reference

1 adjusted for age, smoking, alcohol, TNM stage, treatment and karnofsky performance score; 2 adjusted for sex, age, treatment, karnofsky performance score and comorbidity; 3 adjusted for sex, age, alcohol, treatment, Karnofsky performance score; 4 adjusted for age, TNM stage and karnofsky performance score; 5 adjusted for age, alcohol, TNM stage, second primary tumor and Karnofsky performance score; 6 adjusted for age, smoking, TNM stage and treatment.

An * indicates a p-value < 0·05 and ** indicates a p-value < 0·01.
